# Effect of rearing systems on immune status, stress parameters, intestinal morphology, and mortality in conventional and local chicken breeds

**DOI:** 10.1016/j.psj.2023.103110

**Published:** 2023-09-19

**Authors:** Valentina Stefanetti, Alice Cartoni Mancinelli, Luisa Pascucci, Laura Menchetti, Cesare Castellini, Cecilia Mugnai, Edoardo Fiorilla, Barbara Miniscalco, Diletta Chiattelli, Maria Pia Franciosini, Patrizia Casagrande Proietti

**Affiliations:** ⁎ Department of Veterinary Medicine, University of Perugia, 06124 Perugia, Italy; † Department of Agricultural, Environment and Food Science, University of Perugia, 06124 Perugia, Italy; ‡ School of Biosciences and Veterinary Medicine, University of Camerino, 62024 Matelica, Italy; § Department of Veterinary Sciences, University of Turin, 10095 Grugliasco, Torino, Italy

**Keywords:** alternative poultry system, Italian local breeds, intestinal morphology, cytokine expression

## Abstract

The majority of poultry meat used to be sourced from intensively housed birds. However, consumer preference has since demanded poultry producers develop more sustainable farming systems. Although free-range farming is considered beneficial for animal welfare, it is not as easy to standardize as an intensive system, which makes the choice of bird genotype appear crucial for alternative systems. In this study, we aimed to evaluate the effect of conventional and free-range rearing systems on the immune status, stress parameters, intestinal morphology and mortality in commercial hybrids (Ross 308) and local poultry strains, Bionda Piemontese (**BP**), Robusta Maculata (**RM**), BP x Sasso (**BPxS**), and RM x Sasso (**RMxS**). RNA was extracted from the jejunum and spleen to assess the mRNA expression of *IL-2, IL-6, IL-10, IL-18, IL-1β*, inducible nitric oxide synthase (***iNOS***), toll-like receptor (**TLR**)-4, and interferon gamma (**IFN-γ**). The heterophil:lymphocyte (**H/L**) ratio and intestinal histomorphometric evaluation were also calculated. We found that compared to the conventional system, the rearing system significantly affected the jejunum expression of *IL-10, iNOS, IL-2*, and *IL-6*, where these genes were upregulated in free-range system. A significant interaction between the rearing system and the genotype was also shown. More specifically, local breeds showed a significantly higher expression (*P* < 0.001) of *IL-6* in the free-range system compared to the same genotypes in the conventional system. Moreover, *IL-6* is constantly upregulated in local breeds within the free-range system compared to Ross hybrids. We also found significantly increased H/L and mortality rates in the latter, compared to the local breeds in the free-range reared system. The jejunum morphology also demonstrated a significantly higher villus height in BP and BPxS compared to the Ross hybrids. Overall, the results of our study confirm that the intense selection for growth in broiler chickens may have reduced their ability to react to the environmental stimuli related to free-range systems, resulting in a lower adaptability to a free-range environment, thus making them inappropriate for any farming system other than the conventional one. On the contrary, local chicken breeds are able to adapt and survive in the free-range system of rearing, and represent a genetic resource especially when adaptability to free-range conditions is required.

## INTRODUCTION

Commercial broiler chicken strains are the result of successful selection programs for rapid growth and body conformation (Scheuermann et al., 2003). The main goal of maximizing production efficiency has progressively led to the adoption of intensively housed birds worldwide (Robins and Phillips, 2011). However, in recent years, there has been increasing consumer interest in natural and organic food products. This has affected the poultry industry and enabled alternative rearing systems, for example, organic, agroforestry and free-range systems, to rise in popularity. These production systems provide outdoor runs to allow animals to behave naturally (Jeni et al., 2021). Although the free-range system is considered beneficial for animal welfare, it is not as easy to standardize as the intensive system and the specific risks associated with this alternative type of poultry farming should be taken into account. For example, free-range farming offers poultry flocks more points of pathogen contact via the natural environment (Golden et al., 2021). For the above-mentioned reasons, the choice of bird genotype appears to be crucial for alternative systems, and birds should be selected with good kinetic activity, a well-developed immune system and appropriate body conformation, skeletal development, and growth rates and for their ability to cope with the environment (Sossidou et al., 2015). Castellini et al. (2008) demonstrated that fast-growing strains are unsuitable for an organic rearing system, as they may develop health and welfare problems, for example, leg weakness and high mortality rates due to their excessive weight (Castellini et al., 2008). In addition, Dal Bosco et al. (2010) observed that fast-growing birds tended to remain indoors rather than forage in the pasture, whereas slow-growing birds were more active, spending time outdoors (Dal Bosco et al., 2010).

All these findings suggest that only medium and slow-growing genotypes can fully benefit from an organic rearing system. Some of these strains (e.g., local breeds) could have features or genes, for example, a higher immune response, capable of adapting to extensive rearing systems (Dal Bosco et al., 2021). In such breeds a higher percentage of available resources is employed for metabolic functions other than growth performance, as resistance to heat stress, kinetic activity, and feed research (Cartoni Mancinelli et al., 2020). As EC Regulation No. 889/2008 (Article 8) also stated, the “adaptability” of poultry genotypes to alternative rearing systems has been defined as the key word in establishing that we must take the capacity of animals to adapt to local conditions into account when choosing breeds or strain. According to the Food and Agriculture Organization of the United Nations (**FAO**) (Food and Agriculture Organization of the United Nations FAO, 2023), local breeds of farmed and domesticated animals are at risk of extinction in Europe. Therefore, their conservation is also an important component of poultry biodiversity (Castillo et al., 2021) and should be chosen to promote sustainable, low input, farming systems (Fiorilla et al., 2022).

Thus, it appears critical to find a suitable compromise between animal welfare, breed adaptability to the natural environment, biodiversity protection, and productive performance. This is why an evaluation of growth performance and natural behavior, together with other parameters, for example, gut health, stress, and immunity parameters, should be examined. More specifically, a morphometric evaluation of the small intestine has been considered a good indicator of variations in nutrient digestion and absorption, and a useful tool to evaluate broilers’ overall health and welfare (Biasato et al., 2018). Several studies have reported that the heterophiles:lymphocyte ratio (**H/L**) is influenced by stressors and can be used as a hematological indicator of the stress response in chickens (Salamano et al., 2010). Both the intestinal health of poultry and the H/L ratio are closely related to the housing systems (Shini, 2003; Li et al., 2017) and to the different broiler genetic lines (Campo and Prieto, 2009; Lumpkins et al., 2010). Furthermore, rearing systems and genetics significantly affect the immune gene expression in chickens (Redmond et al., 2010; Yan et al., 2021). Cytokines are essential effector molecules involved in innate and acquired immunity and provide a chicken's first line of defense against several stimuli. Understanding the role of rearing systems and genetics in the expression of immunity-related genes is mandatory for genetic selection in chickens. A broiler population of sires with higher and lower than average proinflammatory cytokine/chemokine mRNA expression levels has been shown to produce progeny with similar profiles (Swaggerty et al., 2008), thus making cytokines a promising tool for genetic selection. Although the choice of chicken genotypes in alternative poultry production is crucial, there remains a lack of studies on the immune and welfare status in poultry under different rearing systems.

The aim of this study was to investigate the effects of genotype and of rearing systems on the immune system (cytokine gene expression), the stress parameters (H/L ratio) and the intestinal morphology and mortality on broilers and on local poultry strains.

## MATERIALS AND METHODS

### Ethic Statement

Chickens were reared according to EU Regulations 834/07, 889/2008, and the Italian directives on animal welfare for experimental and scientific purposes. The Bioethical Committee of the University of Turin (Italy) positively evaluated and approved the experimental protocol with the Protocol Number ID: 251833.

### Animals and Housing

This trial was performed from March to July 2021 at the poultry farm of the Department of Veterinary Sciences of the University of Turin (Italy). A local hatchery purchased 300 male chickens of 5 different genotypes, Bionda Piemontese (**BP**), Robusta Maculata (**RM**), BP x Sasso (**BPxS**), RM x Sasso (**RMxS**), and commercial hybrid (Ross-308). The animals were vaccinated against infectious bronchitis virus, Marek's disease virus, Newcastle disease, Gumboro disease, and coccidiosis and they received food and water ad libitum. The chicks were reared for the first 20 d in the brood, divided into 5 pens, 1 for each genotype. Each pen was 1 m wide and 2 m long and was covered with wood shavings (20 cm deep) as litter and equipped with a waterproof floor and walls. It was environmentally controlled with temperature ranging from 32°C to 20°C and relative humidity (**RH**) from 70 to 65% RH, respectively. At 21 d until slaughter, the birds were randomly divided into 2 different rearing systems: conventional (33 kg of meat per m²) and free-range (21 kg of meat per m²). Three replicates (*n* = 10 cockerels/5 genotypes/2 housing systems/3 replicates) were made for each genotype and system.

In the conventional system, the lighting schedule throughout the trial was 16 h light, 8 h darkness. The environmental conditions in the poultry-house, that is, temperature and RH, were set according to the Ross guidelines (Ross Broiler Management, 2018). No lighting schedule was set for the free-range system and the birds were exposed to a natural temperature and photoperiod. The animals were free to stay inside or outside at any time of the day (at an environmental temperature ranging from 16°C to 26°C). Table 1 shows the 2 different diets used: a starter, administered from 0 to 21 d of age to all birds and grower/finisher, administered from 21 d of age until slaughter (81 d). The diets were analyzed to determine the contents of dry matter, ash, and crude protein by AOAC official methods (AOAC, 2019). The chickens were individually weighed every week in order to establish the appropriate commercial weight (Conventional rearing system: Ross-308 6,200 g; BP 1,690 g; RM 2085 g; BPxS 2,050 g; RMxS 2,230 g. Free-range rearing system: Ross-308 5,800 g; BP: 1,600 g; RM 2,010 g; BPxS 1,980 g; RMxS 2,165 g). The entire performance data are reserved for work currently in progress. Dead birds were recorded daily to calculate the mortality rate and evaluate the causes of death. The birds were slaughtered at a commercial slaughterhouse that provided stunning by electronarcosis, exsanguination by means of an incision of the major blood vessels of the neck, removal of the feathers and hanging of the carcass. Two milliliter blood samples were collected in an EDTA tube to calculate H/L; a 2-cm-long segment of jejunum was removed from each animal, flushed with cold PBS and fixed in 10% neutral-buffered formalin for morphological analysis. Samples of spleen and jejunum were also placed in an RNA*Later* stabilizing solution (Thermo Fisher Scientific, Waltham, MA, USA) and stored at −80°C until biomolecular analyses were conducted.

**Table 1:** Ingredients (%) and chemical composition of starter and grower diets (% f.m.).

Diet	Starter	Grower
Ingredients		
Corn meal	55	58
Soybean meal	25	15
Whole soybean	14	21
Soybean oil	1.5	1.5
Calcium carbonate	1.8	1.8
Monocalcium phosphate	1.3	1.3
Sodium chloride	0.4	0.4
Vitamin/mineral^1^	1.0	1.0
Chemical composition		
Dry matter	89.01	88.88
Crude protein	22.00	19.50
Ether extract	7.22	7.70
Crude fiber	3.04	3.00
Ash	7.04	6.98
Lysine	1.21	1.08
Met. + Cist.	0.72	0.67
Ca/P	1.75	1.81
Estimated metabolizable Energy (Mj/kg)	3,160	3,130

1 Amounts per kg: Vit. A 11,000 U; Vit. D3 2,000 U; Vit. B1 2.5 mg; Vit. B2 4 mg; Vit. B6 1.25 mg; Vit. B12 0.01 mg; α-tocopheryl acetate 30 mg; biotin 0.06 mg; Vit. K 2.5 mg; niacin 15 mg; folic acid 0.30 mg; pantothenic acid 10 mg; choline chloride 600 mg; Mn 60 mg; Fe 50 mg; Zn 15 mg; I 0.5 mg; Co 0.5 mg.

### H/L Ratio Calculation

A blood smear was prepared from a drop of blood without anticoagulant and placed on a single glass slide for each animal. The smears were stained using May-Grünwald and Giemsa stains. A 1:200 Natt–Herrick solution was used to treat the blood samples for the total red and white blood cell counts using an improved Neubauer hemocytometer. One hundred granular (heterophiles, eosinophils, and basophils) and nongranular (lymphocytes and monocytes) leukocytes were counted on the slide and the H/L ratio was calculated (Salamano et al., 2010).

### RNA Isolation and Reverse Transcription

A Quick-RNA Miniprep Kit (Zymo-Research) was used according to the manufacturer's instructions to extract total RNA from 50 mg of ground spleen and jejunum tissue. RNA quantity was calculated by a NanoDrop 2000 Spectrophotometer (Scientific Nanodrop Products, Wilmington, NC) and the integrity of RNA was examined by electrophoresis in a denaturing 1% agarose gel. In order to avoid genomic DNA contamination, 1 µg of total RNA was treated with 1 U of DNase I amplification grade (Invitrogen) and the successful removal of the genomic DNA was confirmed by directly amplifying the RNA extracts without reverse transcription. DNase-treated RNA (500 ng) was reverse-transcribed into cDNA using a mixed priming strategy (Oligo dT + Random Hexamers) with a PrimeScript RT Reagent Kit (Takara) and incubated under the following conditions: 37°C for 15 min and 85°C for 5 s.

### mRNA Cytokine Expression

Table 2 shows the previously published primers used for the relative quantification of target gene expression in the jejunum and spleen tissues: *IL-2, IL-6, IL-10, IL-18, IL-1β*, inducible nitric oxide synthase (***iNOS***), toll-like receptor (***TLR***)-*4*, and interferon gamma (***IFN-γ***). The expression ratio of the genes of interest was normalized according to the abundance of *β-actin* and *GAPDH* as reference genes to adjust for any unbalanced samples and corrected for coexpression in the qRT-PCR. The qRT-PCR reaction was carried out with 5 μL of a 10-fold diluted cDNA in a final volume of 20 μL using 10 μL of SsoFast EvaGreen Supermix (BioRad). Amplification was performed in a CFX96 Touch instrument (BioRad, Hercules, CA) under the following thermal conditions: 98°C for 3 min, followed by 40 cycles of 98°C for 10 s and a different annealing temperature for each primer pair, as reported in Table 2. Each reaction was run in 3 technical replicates and no-template controls (**NTC**) were included in any run. The expression level was normalized using the ΔΔ Cq method (Vandesompele et al., 2002). Following amplification, the specificity of the PCR product was measured by a melting curve analysis and by checking the amplicon size in agarose gel.

**Table 2:** Target gene name, primer sequences, sizes of the amplification products, corresponding accession numbers, primer annealing temperature (*T*_a_) and reference.

Gene name	Primer sequence (5′–3′)	Amplicon size	Accession number	*T*_a_	Reference
*β-Actin*	F: ACGTCGCACTGGATTTCGAGCAGG R: TGCACTCTGTCAGCAATGCCAG	289 bp	NM205518	58°C	Kogut et al. (2012)
*GAPDH*	F: ACTGTCAAGGCTGAGAACGG R: CATTTGATGTTGCTGGGGTC	63 bp	NM204305	56°C	Zhang et al. (2018)
*TLR-4*	F: TGCACAGGACAGAACATCTCTGGA R: AGCTCCTGCAGGGTATTCAAGTGT	335 bp	NM001030693	55°C	Kogut et al. (2012)
*IL-1β*	F: TGGATTCTGAGCACACCACAG R: GACGGGCTCAAAAACCTCCT	155 bp	NM204524	58°C	Haunshi et al. (2017)
*IL-2*	F: TTGGAAAATATCAAGAACAAGATTCATC R: TCCCAGGTAACACTGCAGAGTTT	51 bp	AJ009800	54°C	Kaiser et al. (2000)
*IL-18*	F: GGAATGCGATGCCTTTTG R: ATTTTCCCATGCTCTTTCTCA	264 bp	AJ277865	54°C	Bai et al. (2015)
*IL-6*	F: GCTCGCCGGCTTCGA R: GGTAGGTCTGAAAGGCGAACAG	151 bp	AJ250838	62°C	Swaggerty et al. (2004)
*IFN-γ*	F: GTGAAGAAGGTGAAAGATATCATGG R: GCTTTGCGCTGGATTCTCA	680 bp	Y07922	58°C	Kaiser et al. (2000)
*IL-10*	F: CATGCTGCTGGGCCTGAA R: CGTCTCCTTGATCTGCTTGATG	420 bp	AJ621614	55°C	Rothwell et al. (2004)
*iNOS*	F: TGGGTGGAAGCCGAAATA R: GTACCAGCCGTTGAAAGGAC	240 bp	U46504	55°C	Lammers et al. (2010)

### Intestinal Histomorphological Measurements

The histological examination was conducted with jejunum samples fixed in 10% neutral-buffered formaldehyde and dehydrated in an ascending concentration of ethanol and finally embedded in paraffin. Five micrometer-thick sections were stained with hematoxylin and eosin (Vector Laboratories stain kit). Tissue slices were masked from the treatments and examined under a light microscope (Nikon Eclipse 800) equipped with a digital camera and images were acquired at 4× magnification. Morphometric evaluation was performed by the software Image J (v1.46r, NIH, Bethesda, MD) measuring villus height (**Vh**) and crypt depth (**Cd**). In short, Vh was measured from the villus tip to the villus-crypt junction, whereas Cd was measured from this junction to the base of the crypt; the Vh/Cd ratio was also evaluated (Li et al., 2017). At least 15 intact and well-oriented villi and their associated crypts were measured in each slide.

### Statistical Evaluation

Formal assumptions of the uni- and multivariate approaches were initially verified. Gene expression data for the spleen and jejunum tissues were independently analyzed. Relative cytokine gene expression and intestinal histomorphological examinations were analyzed using a 2-way ANOVA with interaction, in which rearing systems and genetic lines were set as independent variables. Differences were considered statistically significant at *P* ≤ 0.05. Univariate models were analyzed using JASP and GraphPad Prism 9 software and expression values were reported as a mean ± standard deviation. Nonparametric tests were performed on the mortality rate and significance was assessed by the χ^2^ value and set at *P* ≤ 0.05.

A multivariate analysis of cytokine expression in the jejunum tissue and of the H/L ratio was conducted and diagnostic graphs identified ant outliers. Mahalanobis’ distance and studentized residuals were used to identify the outliers for predictors and grouping variables, respectively. Multicollinearity was checked by Tolerance and Variance Inflation Factors. Only variables assessed in 1 tissue (i.e., jejunum, the tissue that showed the largest number of differences between groups) were selected to satisfy the assumption of independence. Among them, variables to be included as predictors in the multivariate analysis were selected to balance the number of predictors and the sample size. Only variables with *P* < 0.1 in the univariate analysis by the *F* test were included in subsequent analyses. To simplify the model, the genetic strains were grouped into 2 categories: fast- (Ross 308) and slow-growing (BP, RM, BPxS, RMxS). Discriminant analysis (**DA**) was used to investigate differences between groups and to identify the variables that contribute most to differentiate these groups. The contribution of each variable to the discriminant function (**Df**) was expressed as structure coefficient, whereas the centroids, indicating the mean discriminant scores of Df for each group, were used to establish the cutting point for classifying samples. Eigenvalues were used to evaluate the relative discriminating power of each Df and the proportion of variance between groups was reported as a further measure of effect sizes. Lastly, Wilks’ lambda was used to test the significance of each Df. The performances of the final Df were estimated by classification statistics (i.e., prediction matrix table) (Pituch and Stevens, 2015; Agradi et al., 2020).

The variables selected by univariate and discriminant analyses were then used to build a dimension describing the immune response of animals using a principal component analysis (**PCA**). A principal component (**PC**) was extracted and items with loadings greater than |0.4| were interpreted. Cronbach's alpha (α) was used to estimate its reliability and values of >0.7 were considered acceptable (Pituch and Stevens, 2015; Menchetti et al., 2020; Cartoni Mancinelli et al., 2021). Corresponding PC scores were calculated by the regression method (Pituch and Stevens, 2015; Menchetti et al., 2020; Cartoni Mancinelli et al., 2021) and analyzed by generalized linear models (**GLMs**). Normal and Identity were set as the probability distribution and the link function, respectively. The effect of the rearing system (2 levels: free-range and conventional), genotype (2 levels: slow- and fast-growing) and their interaction were evaluated, and the least significant difference was used for multiple comparisons. Multivariate analyses were conducted with SPSS Statistics version 25 (IBM, SPSS Inc., Chicago, IL). The level for statistical significance was set at *P* ≤ 0.05.

## RESULTS

### H/L Ratio and Mortality Rate

Table 3 reports the H/L ratio and mortality rate. We found that the H/L ratio was mainly affected by genetic strain (*P* ≤ 0.05) and depended on the strain × rearing system interaction (*P* ≤ 0.05). We observed an increased H/L ratio in all the local breeds reared in a conventional system compared to the free-range birds and Ross strains showed a significant increased H/L ratio compared to the local breeds in controlled housing. The mortality rate was affected by genotype (*P* < 0.05), rearing system (*P* < 0.05), and their interaction (*P* < 0.05). Ross chicken mortality was significantly higher in both rearing systems, especially for free-range birds. The causes of Ross mortality were related to fast growth and the majority of deaths in the conventional system were due to sudden death syndrome (**SDS**) (65%) and ascites (35%), whereas mainly ascites (80%) was reported among the free-range birds. On the contrary, local breeds had a very low mortality rate (0.0–0.5%) in the free-range system.

**Table 3:** Effect of rearing system and genotype on H/L ratio and mortality rate (%) (* *P* ≤ 0.05).

Rearing system	Genotype	H/L	Mortality rate (%)
	BP	0.32	0.0
	RM	0.41	0.5
Free-range	BPxS	0.35	0.0
	RMxS	0.40	0.0
	Ross	0.82	3.9
	BP	0.42	1.2
	RM	0.44	1.4
Conventional	BPxS	0.46	0.9
	RMxS	0.64	1.7
	Ross	0.62	3.0
		Significance	
Rearing System		NS	(chi-square)*
Genotype		*	*
Interaction		*	*

### Cytokine Expression Profile

The rearing system did not affect the cytokine gene expression in the spleen, except for *IL-2*, which was significantly higher (*P* < 0.05) in the free-range system compared to the conventional one (Supplementary Figure 1). No significant interaction between genotype and rearing system was observed in the spleen. In this tissue, the genotype had an effect only on *IL-10*, which was significantly higher in all the local breeds (*P* < 0.001) when compared to fast-growing broilers.

**Figure 1:**
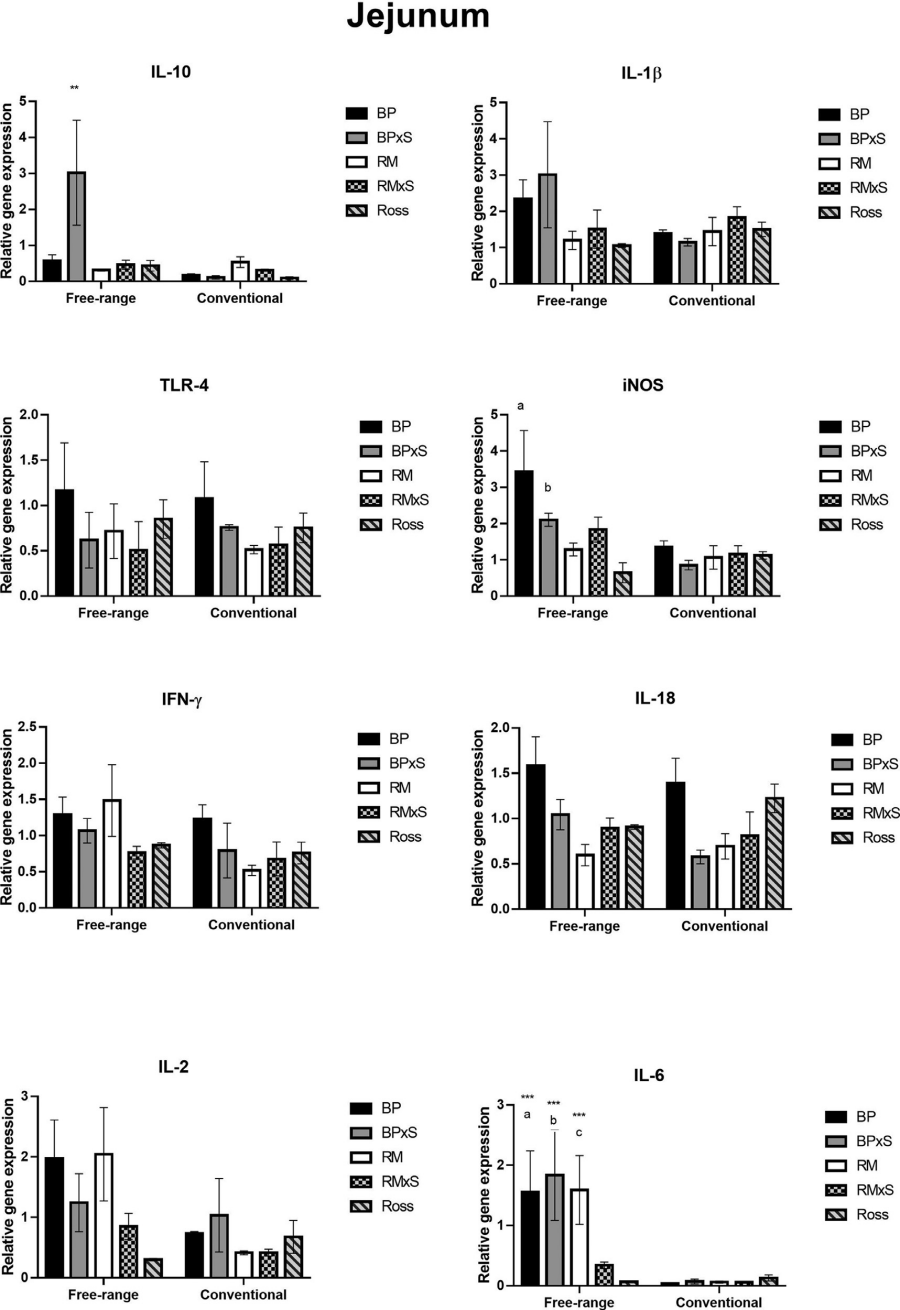
Gene expression analysis in jejunum tissue. Each value was normalized to the amount of 2 reference genes (*ACTB* and *GAPDH*) and was exhibited as means of all the triplicate experiments ± standard deviation. Technical replicates were averaged and only those samples with a standard error lower than 0.2 Cq were maintained. Asterisks indicate a significant increase in the cytokine expression between the same genotype in the 2 rearing systems. *P < 0.05, **P < 0.01, ***P < 0.001. Letter (a) indicates a statistically significant difference within the same rearing system, between BP and Ross; (b) between BPxS and Ross; (c) between RM and Ross.

Figure 1 shows cytokine expression in the jejunum tissue. The rearing system had a significant effect on the expression of *IL-10, iNOS, IL-2*, and *IL-6*, where these genes were upregulated in the free-range system compared to the conventional system. Significant interaction between the rearing system and the genotype was also displayed. More specifically, BPxS had a higher expression of *IL-10* (*P* < 0.001) in the free-range system compared to the same genotype in a conventional system. BP, BPxS, and RM showed significantly higher expression (*P* < 0.001) of *IL-6* in the free-range system compared to the same genotypes in the conventional system. Moreover, *IL-6* was constantly upregulated in BP, BPxS, and RM compared to Ross in the free-range system. BP and BPxS showed a higher *iNOS* expression (*P* < 0.05) compared to the free-range reared Ross. No significant differences or interactions were observed in the expression of *IL-18, IL-1β, IFN-γ*, and *TLR-4.*

### Multivariate Analysis

**Figure 2:**
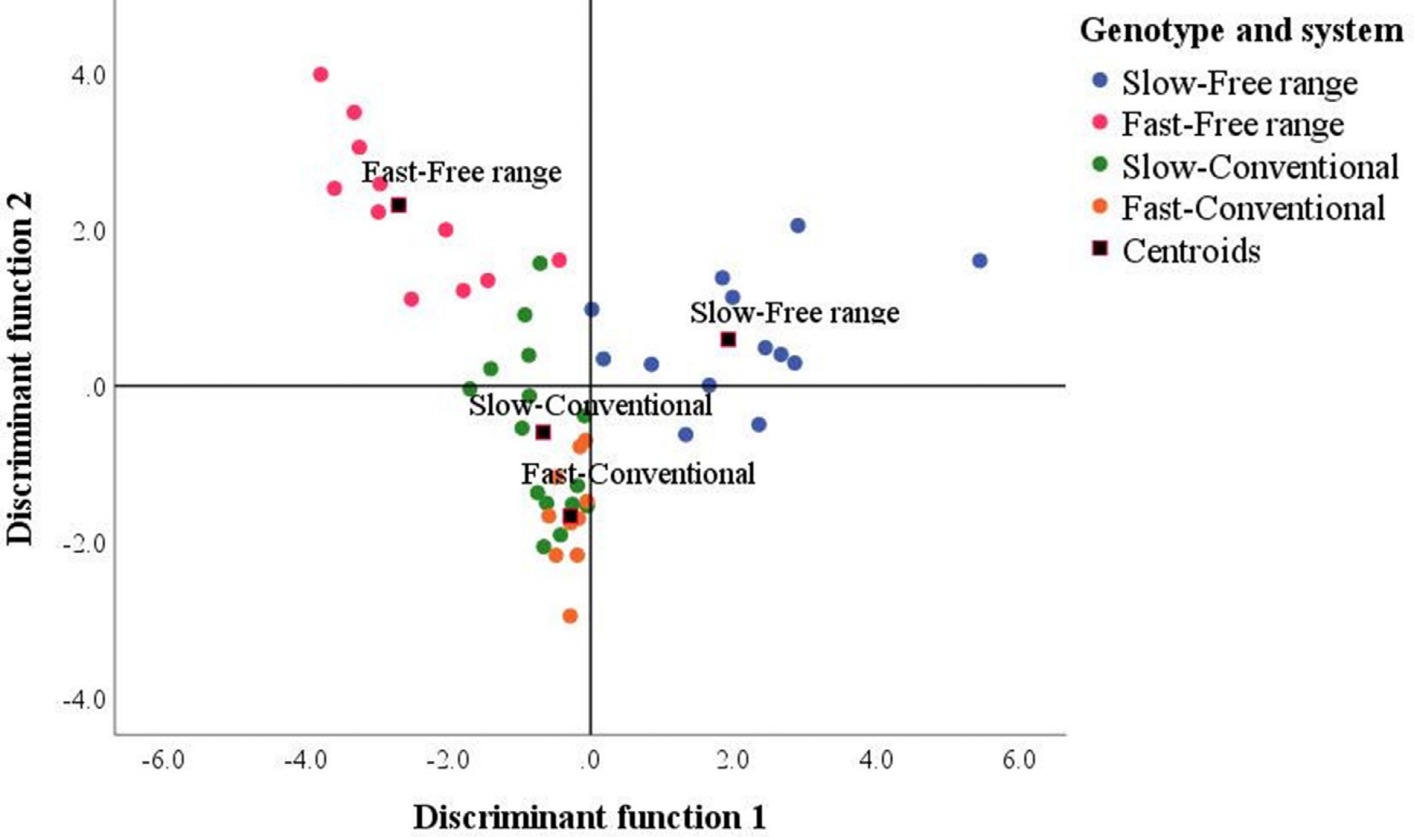
Scatterplot of discriminant function (Df) scores according to the group and group centroids. Differences in Df1 scores (along the *x*-axis) mostly indicated changes in the values of *IL-6, IL-2*, and *iNOS* while differences in Df2 scores (along the *y*-axis) mostly indicated changes in the values of H/L and *IL-10*. Centroids are the mean discriminant scores for each group.

A multivariate analysis enabled the combined relation between the different traits to be considered. Table 4 shows the 5 variables selected as predictors for DA. The accuracy of the DA was 87.1%. The misclassified cases were samples of the slow growing, reared in free range (erroneously included in the slow-conventional group). Overall, 11 values were excluded as univariate outliers, whereas diagnostic measures did not indicate any multivariate outliers or multicollinearity issues. Table 4 also shows the structure matrix and the effect size of each Df. The majority of the differences between the groups were explained by the first 2 Dfs. Df 1 included *IL-6, IL-2*, and *iNOS* with positive loadings and H/L with a negative loading, whereas Df 2 included H/L and *IL-10*, both with positive loadings. The values of eigenvalue and Wilk's lambda indicated that Df 3 did not play a significant discriminatory role and, therefore, it was not interpreted and analyzed further. As regards Df1, the centroid (i.e., the mean discriminant scores for each group; Supplementary Table S1 and Figure 2) of the fast free-range group (−2.691) is much smaller than that of the slow free-range group (1.927), whereas the 2 conventional groups have similar centroids for this Df (−0.664 and −0.286 for slow and fast conventional groups, respectively). This indicated that the Df1 (*x*-axis) mainly discriminated the response of the 2 groups of genotypes reared free-range. In this system, slow-growing birds had positive scores (i.e., high *IL-6, IL-2*, and *iNOS* concentrations and low H/L), whereas fast-growing birds had negative scores (i.e., low *IL-6, IL-2*, and *iNOS* concentrations and high H/L). In Df2, the greatest difference between centroids was found between the fast free-range group (2.318) and the fast conventional group (−1.670). This suggests that Df2 (*y*-axis) mainly characterized the responses of the fast-growing broilers to the rearing system: the latter had positive scores (i.e., high H/L and *IL-10*) in the free-range system and negative scores in the conventional system.

**Table 4:** Structure matrix and effect size of each discriminant function.

Variable/parameter	Function		
	1	2	3
*IL-6*	**0.660**	0.428	−0.165
*IL-2*	**0.623**	0.178	0.045
*iNOS*	**0.576**	0.065	0.095
H/L	−0.519	**0.703**	−0.299
*IL-10*	0.155	**0.457**	0.318
Eigenvalue	2.447	1.144	0.302
Proportion of variance between groups	71.0%	53.3%	23.1%
Significance of Wilk's lambda	<0.001	0.004	0.159

In bold the largest absolute correlation between each variable and any discriminant function.

**Figure 3:**
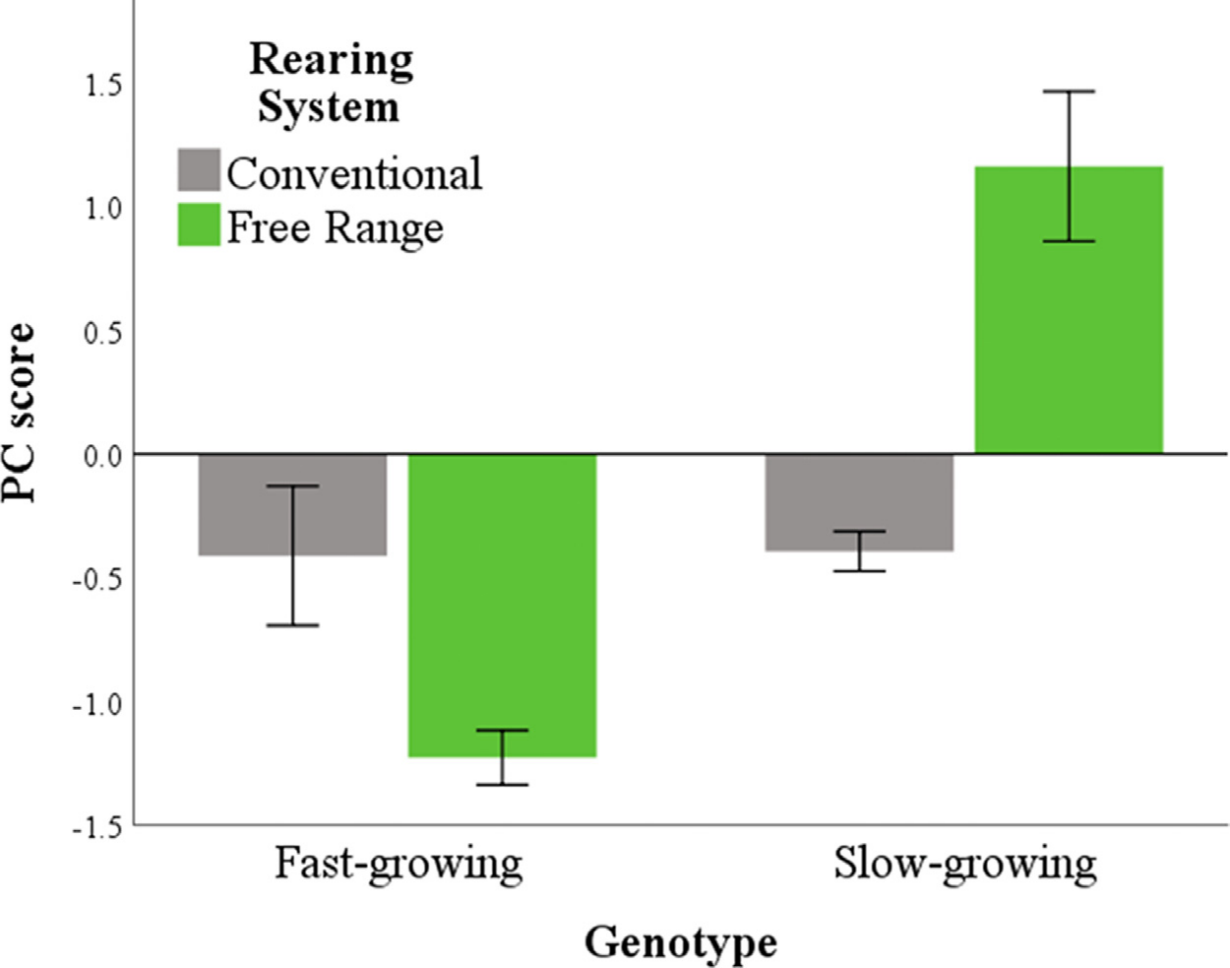
Principal component (PC) scores according with rearing system and genotype. Values are reported as means ± standard errors.

Variables included in the first 2 Dfs were then selected for the PCA (Table 5). One bipolar PC was extracted, including *IL-2, IL-6*, and *iNOS* with a positive loading and H/L with a negative loading. The low coefficient of *IL-10* indicated the low contribution of this variable to the PC which, in turn, showed an acceptable, internal consistency (α > 0.7). The PC scores (Figure 3) were influenced by the genotype (*P* < 0.001), which interacted with the rearing system (*P* < 0.001). Fast-growing broilers had negative scores in conventional systems, which tended to further reduce when they were reared in the free-range system (*P* = 0.062). Negative PC scores indicated low values for *IL-2, IL-6*, and *iNOS* and high values for H/L. Slow-growing birds, on the other hand, had negative scores in the conventional system, although they significantly increased in the free-range system (*P* < 0.001). The increase in the PC scores indicated an increase in *IL-2, IL-6*, and *iNOS* values increased, whereas the H/L values decreased.

**Table 5:** Factor loadings for the variable included in the principal component and measure of its reliability (Cronbach's α).

Item	Principal component
*IL-2*	**0.907**
*IL-6*	**0.867**
*iNOS*	**0.763**
H/L	**−0.545**
*IL-10*	0.365
Cronbach's α	0.748

Factor loadings with an absolute value greater than 0.4 are in bold.

### Jejunum Histomorphology

Table 6 summarizes the effects of the rearing system, genotype and their interaction on the jejunum histomorphology. In our study, the rearing system significantly affected the Vh and Vh/Cd ratio, showing higher values (*P* < 0.001) in BP and BPxS reared in the free-range system compared to the same genotype in the conventional system. Moreover, within the free-range rearing system, BP and BPxS showed significantly higher values of Vh compared to Ross. All the local breeds reared in the conventional system showed a significantly lower Cd (*P* < 0.001) and higher Vh/Cd ratio (*P* < 0.01) compared to Ross.

**Table 6:** Effect of rearing system and genotype on histomorphology of jejunum.

Rearing system	Genotype	Vh (µm)	Cd (µm)	Vh/Cd
	BP	1079.10	257.44	4.46
	RM	896.09	207.98	4.16
Free-range	BPxS	1247.40	302.38	4.49
	RMxS	985.82	304.04	3.09
	Ross	954.50	238.80	4.11
	BP	390.20	175.07	5.31
	RM	882.49	231.72	4.11
Conventional	BPxS	842.43	289.90	2.95
	RMxS	947.93	250.65	4.03
	Ross	893.68	623.80	1.52
		Significance		
Rearing system		***	NS	***
Genotype		**	***	***
Interaction		**	*	**

Vh, villus height; Cd, crypt depth; Vh/Cd villus height/crypt depth ratio. NS, not significant; *P < 0.05; **P < 0.01; ***P < 0.001.

## DISCUSSION

Cytokines are finely regulated and play a key role as communication signals. *IL-2* has an important role in the immune response to antigenic stimuli; *IL-1β* and *IL-6* are produced by monocytes and macrophages and act as proinflammatory cytokines with a relevant role in early phases of inflammation by eliminating pathogens from the host. *IL-10* is a pivotal anti-inflammatory cytokine, able to inhibit proinflammatory cytokines. When proinflammatory cytokines are expressed in large quantities, the host can regulate the inflammatory response by upregulating the expression of anti-inflammatory cytokines. This results in positive feedback to maintain immunity balance (McGeachy et al., 2007; Ding et al., 2021), and avoiding the intensification of a proinflammatory response.

In this research, we found that in spleen tissue, the rearing system has an impact on *IL-2* expression, which is constantly upregulated in free-range compared to the conventional system. Interestingly, *IL-10* is overexpressed at spleen level in all the local breeds compared to commercial, fast-growing broilers, thus suggesting a “breed-effect” for this proinflammatory cytokine. We found a significantly elevated expression of *IL-10* in jejunum tissue in BPxS and *IL-6* in BPxS, BP and RM, reared in a free-range system compared to the same conventionally housed genotypes. These results could be due to the fact that free-range systems are characterized by environmental conditions, where poultry are potentially exposed to a wide range of naturally present intermediate hosts or stressors in the flock (Jeni et al., 2021). Some authors investigated the expression of proinflammatory cytokines in the intestinal tract of broilers in net, floor, and cage rearing systems, and discovered significantly lower levels in broilers raised in cages. They concluded that, even though cage rearing prevents birds from direct contact with all the environmental stressors, it does not afford the birds any microbe priming effects for the immune system, or the benefits resulting from litter material ingestion. The consequence may be poorer adaptability compared to broilers reared on floors and in pens (Yan et al., 2021). In this study, we also found that within the free-range rearing system, the BPxS, BP, RM showed significantly higher expression values of *IL-6* and *iNOS* compared to the Ross 308. This could be due to the resource allocation theory, which states that the body energy of fast-growing animals is mainly allocated for growth and maintenance and therefore, the amount required to mount an immune response could be quite insufficient (Humphrey and Klasing, 2004). This speculation has also been confirmed by the growth performance results (data not reported here). The individual live weight for Ross showed higher values compared to local breeds and their crossbreed in both conventional and free-range systems (*P* < 0.001). The resource allocation hypothesis is also consistent with our results on the H/L ratio of Ross chickens, which were higher than the results for the local breeds in both housing systems. Heterophils and lymphocytes are the 2 most abundant white blood cell types in birds, which play an essential role in innate and adaptative immunity, respectively (Minias, 2019). Stress increases heterophiles and reduces lymphocytes (Mosca et al., 2019), and H/L is considered a disease resistance trait (Thiam et al., 2022). Therefore, the increased H/L we observed in the free-range reared Ross is indicative of a state of stress and a reduced ability to cope with microbial diffusion and pressure (Minias, 2019) in this housing system. Our study appears to confirm that the intense selection for growth in broiler chickens may have reduced their ability to react to the environmental stimuli found in free-range systems. This results in a lower adaptability to the free-range environment and makes them inappropriate for any farming system other than a conventional system. The greater incidence of SDS in the conventional system compared to the free-range system could be due to a slower growth rate of these birds in a free-range system (increased needs for thermoregulation, and other activities in uncontrolled environmental conditions + increased energy costs due to a lower animal density and higher kinetic activity) (Castellini et al., 2002; Bergmann et al., 2017). On the contrary, local breeds presented a reduced H/L ratio and lower to absent mortality rate in the free-range system, thus implying a better, more appropriate resilience of these animals to this type of rearing system (Dal Bosco et al., 2021).

The multivariate analysis also demonstrated that slow-growing birds in the free-range system had positive scores (i.e., high *IL-6, IL-2*, and *iNOS* expression and low H/L ratio), whereas the fast-growing birds had negative scores (i.e., low *IL-6, IL-2*, and *iNOS* expression and high H/L ratio), which confirmed that local breeds are characterized by a certain degree of adaptability toward the environmental conditions. Several authors have conducted a comparative analysis between indigenous and exotic ovine (Yang et al., 2021) and chickens breeds (Haunshi et al., 2017) in order to investigate their adaptability to environmental stressors by means of cytokine expressions. Yang et al. (2021) demonstrated the levels of IgG and *IL-1β* were significantly higher in indigenous Kazakh sheep compared to Suffolk sheep, which grow quickly, but do not adapt to harsh environments and have lower levels of IgG and cytokines. Hence, they concluded that indigenous breeds may have a higher immunity and adapt more easily to stringent environmental conditions compared to Suffolk sheep (Yang et al., 2021). Previous studies have demonstrated a higher expression of *TLR-4, TLR-5*, and *TLR-7* in the indigenous chickens of India compared to the White Leghorn (Ramasamy et al., 2010; Haunshi et al., 2017). TLR-4 is the principal receptor for LPS, the main component of the outer membrane of gram-negative bacteria. It is required for LPS-mediated inducible nitric oxide synthase (**iNOS**) induction in chicken macrophages (Dil and Qureshi, 2002). Hence, the significantly higher expression of *TLR-4* has implications in nitric oxide production and innate immunity. TLR-5 recognizes flagellin, the main component of bacterial flagella. Thus, the higher expression of *TLR-5* could provide better protection against bacteria with flagella as a major, pathogenic component. Lastly, TLR-7 has been implicated in the intracellular recognition of nucleic acids. Furthermore, the higher *TLR-7* expression might provide protection against viral diseases. For all the above-mentioned reasons, the authors of these studies concluded that indigenous breeds are hardy and are able to more easily adapt to and survive in the unfavorable conditions of free-range or extensive rearing systems compared to exotic, improved breeds (Ramasamy et al., 2010; Haunshi et al., 2017). Interestingly, we found that the interaction between genotypes and the rearing system has a significant impact on jejunum histomorphometric analysis. Intestinal morphology traits, including villus height, crypt depth, and Vh/Cd ratio, are indicative of a bird's gut health, nutrient digestion, and absorption capacity. Increased villus height and Vh/Cd ratio and decreased crypt depth are directly associated with a greater ability to absorb nutrients (Ding et al., 2021). In our study, we found BP and BPxS reared in free-range system have significantly higher villi compared to the same genotype in a conventional system. In the free-range rearing system, BPxS and BP also showed significantly higher values of Vh compared to Ross. The longer the Vh in their small intestine, the greater their ability to absorb nutrients (Caspary, 1992; Awad et al., 2009). Birds in a free-range system ingest fibrous plant material, insects and worms, which have a high fiber concentration and other substances that positively affect nutrient digestion, gut morphology (Awad et al., 2009), and cecal microbiota and the production of short-chain fatty acids (Borrelli et al., 2017). The latter, together with the strong aptitude of the local breeds for foraging could explain our results.

Based on genotype, all the local breeds reared in the conventional housing system showed significantly a lower Cd and higher Vh/Cd compared to Ross. This lower ratio indicates possible injuries of the mucosa and reduced digestion/absorption (Ding et al., 2021). The improvement we found in local breeds reared outdoors may be associated with a more balanced microflora, which plays a role in intestinal morphology. The intestinal tract harbors a complex ecosystem of bacteria to maintain gut homeostasis. *Lactobacilli* and *Bifidobacteria* are regarded as beneficial for modulating the gut's immune response, by forming a mucosal barrier to protect the gut from harm (Li et al., 2017). The increased population of these bacteria in the jejunum could inhibit the colonization of pathogens, such as coliforms, by producing specific compounds, for example, bacteriocins and organic acids, which reduces the incidence of their adverse effects on the intestinal mucosa (Jazi et al., 2017; Teymouri et al., 2021). In order to better explore and confirm this hypothesis, further microbiome analyses are required.

## CONCLUSIONS

It is crucial to direct efforts toward identifying a genotype characterized by a certain degree of adaptability toward the free-range environment. Understanding the relation between innate immunity (cytokine profile), the stress parameters (H/L ratio), and gut health (histomorphological evaluations) could be useful for finding strategies for the conservation and genetic improvement of local breeds. These breeds are in danger of extinction worldwide due to their replacement by more productive poultry strains. However, in addition to conserving biodiversity, they might represent a genetic resource of high-quality products, especially when adaptability to free-range conditions is required.

In conclusion, our work suggests that both genotype and the rearing system play a role in the animal's immune response and welfare. Local breeds are able to cope with the extensive system better than the fast-growing, commercial strains, thus making the former an important genetic resource for alternative production systems. Adaptability to organic and free-range systems involves multiple parameters. Therefore, in addition to the daily weight gain, behavior, and welfare, other aspects related to immunity, stress, and gut health should be taken into account in order to establish a viable adaptability index.

## Appendix Supplementary materials

Image, image 1

Image, application 2

## Footnotes

Supplementary material associated with this article can be found in the online version at doi:10.1016/j.psj.2023.103110.
