# Supplementary figures and images for: Effect of rearing systems on immune status, stress parameters, intestinal morphology, and mortality in conventional and local chicken breeds
Source: Poult Sci. 2023 Sep 19;102(12):103110. doi: 10.1016/j.psj.2023.103110 (PMC10591014; doi:10.1016/j.psj.2023.103110)

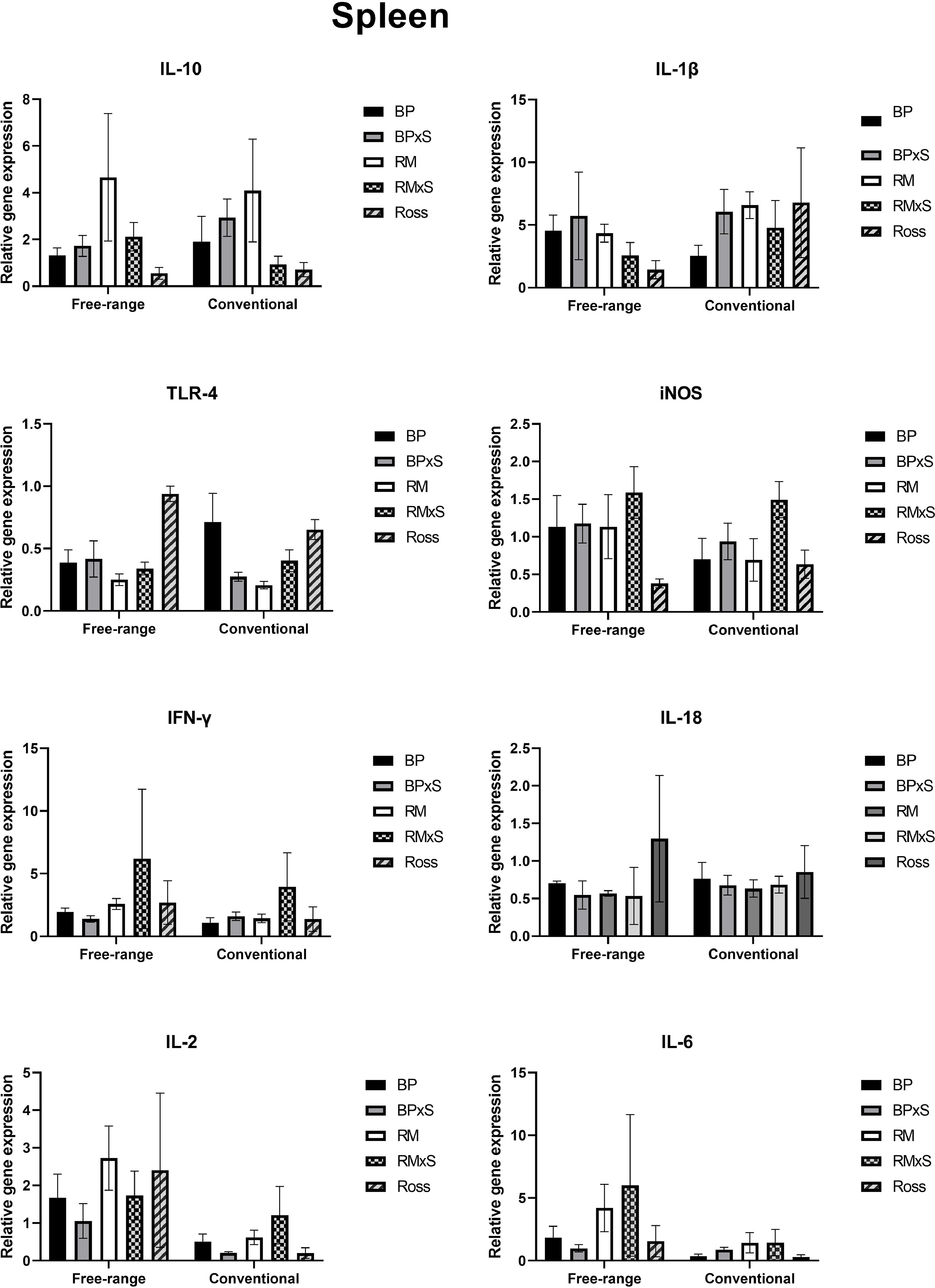

Supplement: Supplementary file 1 [file mmc1.jpg]
