# Supplementary material for: Effect of rearing systems on immune status, stress parameters, intestinal morphology, and mortality in conventional and local chicken breeds
Source: Poult Sci. 2023 Sep 19;102(12):103110. doi: 10.1016/j.psj.2023.103110 (PMC10591014; doi:10.1016/j.psj.2023.103110)
Supplement: Supplementary file 2 [file mmc2.docx]

**Supplementary material**

**Table S1. Functions at group centroids of the discriminant functions**

| **Genotype and system** | **Function** | | |
| --- | --- | --- | --- |
|  | **1** | **2** | **3** |
| Slow-growing Free range | 1.927 | 0.593 | -0.038 |
| Fast- growing Free range | -2.691 | 2.318 | -0.379 |
| Slow- growing Conventional | -0.664 | -0.596 | 0.314 |
| Fast- growing Conventional | -0.286 | -1.670 | -1.752 |
